# Exploring the Potential Toxicological Mechanisms of Vine Tea on the Liver Based on Network Toxicology and Transcriptomics

**DOI:** 10.3389/fphar.2022.855926

**Published:** 2022-03-22

**Authors:** Fangyu Xiao, Jihua Qiu, Ying Zhao

**Affiliations:** ^1^ Guangzhou University of Chinese Medicine, Guangzhou, China; ^2^ South China Agricultural University, Guangzhou, China; ^3^ Department of Gynecology, First Affiliated Hospital of Guangzhou University of Chinese Medicine, Guangzhou, China; ^4^ First Clinical Medical College of Guangzhou University of Chinese Medicine, Guangzhou, China

**Keywords:** vine tea, hepatotoxicity, network toxicology, molecular docking, molecular dynamics, GEO database

## Abstract

**Objective:** This study focuses on whether vine tea contains potentially toxic components that trigger hepatotoxicity as a mechanism of action, which further provides some reference for the consumption and guides future product development of vine tea.

**Methods:** The chemical components of vine tea were collected from the reported literature and the toxicological information matched with the CTD database was collected, and the dataset of potential toxic components was established. The toxic components were submitted to the PharmMapper server to obtain potential targets. At the same time, the relevant targets were searched in the CTD database and GeneCards database with keywords such as “Hepatic Toxicity,” “Liver Damage,” and “Drug-induced liver injury.” After intersection, the potential hepatotoxic targets of vine tea were obtained. The protein interactions of potential hepatotoxic targets of vine tea were analyzed by the STRING database. Protein–protein interaction (PPI) networks were constructed by Cytoscape3.6.1 software. The GO molecular function and KEGG pathway of hepatotoxic targets were enriched by the R package to screen the key targets. The role of the components and key targets was analyzed by the LEDOCK program. The data from GEO database were mined for the functional correlation characterized by cell transcriptional expression caused by vine tea as a disturbance factor.

**Results:** This study has searched 34 potential toxic components and 57 potential hepatotoxic targets of vine tea, and the result showed that these targets were mainly involved in oxidative stress, cell metabolism, and apoptosis to affect the liver.

**Conclusion:** Vine tea has the interrelationship of multi-components, multi-targets, and multi-pathways. At the cellular level, the toxic components of vine tea, mainly flavonoids, may promote oxidative stress, promote oxidation to produce free radicals, guide apoptosis, and affect cell metabolism and other cytotoxic mechanisms. However, this hepatotoxicity is related to the dose, duration of vine tea, and individual differences. This study revealed the potential hepatotoxic components of vine tea and provides a reference for further research and development of related functional products.

## Introduction

Vine tea is made from the tender leaves of *Ampelopsis grossedentata* (Hand. -Mazz.) W.T. Wang, a genus of *Ampelopsis* in the family Vitaceae, and the chemical composition of vine tea is mainly flavonoids and polyphenolic substances (Jia et al., 2021), with a total flavonoid content of up to 40% or more (He et al., 2000), represented by dihydromyricetin (30%) (Wang et al., 2014). Vine tea has been classified as a new food resource by the Chinese regulatory authority since 2013 and is generally taken orally as a raw herb for medicine. Vine tea is a typical tea-like plant in which the tender leaves are simply stir-fried during production to inhibit the action of intracellular enzymes, thus maximizing the retention of endogenous components of vine tea. Vine tea has been consumed in China for thousands of years and is considered a therapeutic tea or herbal tea in the Tujia, Yao, and Hakka regions of China for anti-inflammatory purposes (Tong et al., 2020), treatment of sore throat (Gao et al., 2009), and prevention of high fat (Fan et al., 2020).

There has been a misconception that Chinese herbal medicines are natural and less toxic, whereas chemically purified drugs are more toxic, when in fact, several have found that Chinese herbal medicines cause drug-induced liver injury (Sun et al., 2018; Zhao et al., 2018); that is, the chemical components of Chinese herbal medicines or their metabolites have toxic effects on the liver, and clinical data show that 42.9% of drug-induced liver injuries are caused by Chinese herbal medicines (Fengqin et al., 2011).

Presently, vine tea is becoming more popular among consumers worldwide because of its therapeutic functions and taste, but the assessment of its potential toxicity and drug interactions has not been definitional, and only few components are considered safe for consumption, such as dihydromyricetin (Zhang et al., 2021), so it is debatable whether vine tea has potential toxicological effects on humans after consumption, for it contains rich flavonoids. In the past two years, a case of a patient with multi-organ functional impairment due to vine tea poisoning admitted to the First Affiliated Hospital of Guangxi Medical University was reported (Faxin et al., 2020), which shows that the research on the mechanism of hepatotoxic effects of vine tea should be paid attention.

However, the present research on vine tea mainly focuses on chemical composition, nutritional composition, and pharmacology, and only some studies on toxicology are available. Moreover, traditional toxicological studies generally use animal models as the basis and pathology and immunology as the techniques to study the toxic targets of drugs, which not only have a long experimental period and high cost but also the correlation of the studied toxic targets is poor and it is more difficult to summarize the core targets. Until the concept of network toxicology (Fan et al., 2011) was proposed, the potential core targets, GO molecular functions, and KEGG signaling were analyzed by constructing herbal–component–target networks and combined with molecular docking techniques to verify the interactions between vine tea components and important targets, as well as by mining gene chips or RNA-seq to analyze the functions characterized by the transcriptional expression of vine tea components as perturbation factors. This study mainly used network toxicology and transcriptomics to analyze the potential toxic components of vine tea and reveal the potential mechanism of action for the occurrence of hepatotoxicity in vine tea, which further provides some reference for the consumption and guides future product development of vine tea.

In this study, first, a dataset of potentially toxic components of vine tea was established and these components were used to obtain binding targets through the PharmMapper server. On the other hand, the CTD database and the GeneCards database were searched to obtain targets with high correlation with keywords, such as “Hepatictoxicity,” “Liver Damage,” and “Drug-induced liver injury.” Next, potential hepatotoxic targets were obtained after the intersection of these two kinds of target collections. Then, the protein–protein interaction network was constructed for the aforementioned obtained hepatotoxic targets and acquiring the key targets with the toxic components of vine tea by combining GO and KEGG analysis. Finally, the relationships between the relevant toxic components of vine tea and the key targets were verified by a molecular docking technique and dynamics simulation. Subsequently, the RNA-seq expression profiles acquired from the experiment with hepatotoxic components as perturbation factors were further subjected to an enrichment analysis of the biological process (BP) module to observe the differences between the enrichment analysis results and the biological processes involved in the toxic targets acted by vine tea.

## Materials and Methods

### Establishment of the Dataset of Toxic Components of Vine Tea

The domestic and foreign literature (Wang et al., 1999; Du et al., 2004; Ming et al., 2015; Vieira Carneiro, 2016; Gao et al., 2017) was looked up and the chemical composition of vine tea was collected, and the chemical composition information was matched with the PubChem database (Kim et al., 2021) to obtain the 3D stereo structure and SMILES numbers of the compounds. The SMILES numbers were entered into the SWISS database (Daina et al., 2017) for predicting the ADMET properties of the compounds, and the compounds with intestinal absorption and compliance with Lipinski’s rule were screened as potential active components. The toxicological information of the active components was further queried through the CTD database (Davis et al., 2021) to establish a dataset of potentially toxic components of vine tea.

### Prediction of Potential Targets of Vine Tea and Acquisition of Liver-Related Targets

The 3D stereo structure of vine tea toxicity components dataset was submitted to the PharmMapper server (Wang et al., 2017) for the prediction of relevant action targets. The prediction results were matched with the UniProt database (UniProt, 2021) to filter human-derived targets and annotate the target information as official symbols to obtain the potential action target set A of vine tea toxicity components. These keywords, such as “Hepatictoxicity,” “Liver Damage,” and “Drug-induced liver injury,” were imported into the GeneCards database (Stelzer et al., 2016) and CTD database to obtain the relevant targets of hepatotoxicity. The targets with a score value greater than 200 in the returned results of the GeneCards database and those with reported direct evidence in the returned results of the CTD database were used as filtering conditions, and the hepatotoxicity target set B was further established by deleting duplicate and false-positive genes. The predicted potential targets of vine tea toxicity components set A and the hepatotoxicity target set B were intersected to obtain the potential hepatotoxicity targets of vine tea toxicity components.

### Construction of Protein–Protein Interaction Network for Hepatotoxic Targets

The potential hepatotoxic targets of vine tea were analyzed by the STRING online server (Szklarczyk et al., 2020) for protein–protein interactions (PPIs) and importing the results into Cytoscape 3.6.1 software for visualization analytics. CytoHubba and NetworkAnalyzer plug-ins were used to calculate the nodes and edges of the entire PPI network. First, the degree calculated by the CytoHubba plug-in was demonstrated by node color. Second, the degree of closeness centrality calculated by the NetworkAnalyzer plug-in was represented by node size. Third, the degree of the interaction between the nodes was represented by a circular-ranked approach which represents a further analysis of the importance of nodes in the network and key target proteins.

### GO Enrichment Analysis and KEGG Pathway Enrichment Analysis

An enrichment analysis of GO molecule function and KEGG pathway was performed by the Bioconductor (www.bioconductor.org/) bioinformatics analysis package of R software (https://www.r-project.org/). Running the R 4.0.2 software, the R package (org.Hs.eg.db) was referenced, transforming potential hepatotoxic target gene names of vine tea toxic components for gene ID numbers, and the GO biomolecular functions and KEGG pathways of the targets were enriched by the clusterProfiler (Yu, 2018).

### Molecular Docking and Molecular Dynamics Simulation

The top 25 important nodes of the PPI network and the genes with corrected *p* < 0.01 for KEGG pathway hits were used as filtering criteria to screen key target proteins for potential hepatotoxicity of vine tea. Protein crystal structures with more comprehensive information were searched in the PDB database (Burley et al., 2021), with human, inclusion of natural ligand molecules, and resolution ≤3.5 Å as the priority criteria for screening, and the stereo structures of protein crystals were downloaded. The LEDOCK program (Zhang and Zhao, 2016) was used to perform a semi-flexible molecular docking of vine tea toxic components with key target proteins, which the docking program set the receptor protein as a rigid molecule and the ligand as a flexible molecule, and the lowest energy conformation was further searched using simulated annealing and genetic algorithm crossover operations. Compared with the natural ligand of the protein crystal and the ligand with a significant effect reported in the literature, the RMSD value of the natural ligand after docking should be less than 2 Å to verify the accuracy of the docking program parameter settings. The output file of the docking task was sorted by the energy space conformation of ligands with a score system, and selecting the top-ranked group of receptors and ligands for molecular dynamics simulations to verify the reliability of the docking procedure. Using the lowest energy conformation after docking as the input file for molecular dynamics simulations, GROMACS software was used to perform fully flexible simulations of the protein and ligand molecules to further analyze the interaction between the protein and ligand molecules in the aqueous solvent system. The topology file of the protein and ligand molecules was constructed using the CHARMM36 force field (Huang et al., 2017), a dodecahedral unit box was defined for periodic simulations. Afterward, the water model of the TIP4P force field was used as the solvent to fill the unit box, while chloride or sodium ions were added to make the whole system neutral. Before running the kinetic simulation, the whole system was energy minimized, the system temperature was controlled to 300 K and pressure to 1bar, and after the system passed the NVT and NPT system equilibrium, the molecular dynamics simulation would be run for ten ns.

### Transcriptome Analysis

The aforementioned analysis would obtain the important hepatotoxic components of vine tea. The expression profiles of the relevant microarray and RNA-seq were searched and downloaded from the GEO database using the components as perturbation factor, when expression profiles of compounds that have been reported to be hepatotoxic as perturbation factors were also obtained as reference. The expression profiles of different perturbers were grouped and analyzed for gene expression. Based on the highly conserved gene expression profiles of different species (Alam et al., 2020), if the expression profiles were for nonhuman species, the homologous transformation of genes was performed through the Ensembl database. The expression profiles were further subjected to a gene set enrichment analysis (GSEA), using the biological process (BP) of Gene Ontology (GO) as a functional module for enrichment, and the process was implemented through the gseGO of clusterProfiler. The enrichment results were obtained GO functions with significant levels by screening conditions of *p* < 0.05 and enrichment score >50. The GO functions enriched with vine tea components as perturbation factors were compared with the GO functions of reference expression profiles for similarity, respectively, and the correlation between GO functional modules was analyzed by the GOSemSim package. The similarity comparison used the GO function similarity algorithm explored by Wang et al. (2007). Generally speaking, the correlation of 0.6–0.8 indicates that both are similar, and the correlation >0.8 is considered as homology. The GO functions of vine tea components as perturbation factors and the GO function correlation >0.6 of the reference expression profile were screened as functional modules with similarity. The GO functions related to liver injury were selected for analysis. Hit gene sets corresponding to the similar modules were compared for gene function similarity to verify the functions characterized by transcriptional expression of different components of vine tea after perturbation of cells.

## Results

### Dataset of Toxic Components of Vine Tea

Searching the reported literature relating to vine tea, a total of 95 chemical components were collected, mainly including flavonoid, flavonoid glycoside, isoflavone, limonoid, fatty acid, and volatile component. The ADMET properties of the chemical components were predicted by the Swiss database, and 60 active components that conformed to human intestinal absorption and Lipinski’s rule were filtered out. Moreover, matching the toxic information of compounds in the CTD database, and 34 toxic components with direct evidence were screened out. The obtained information of potential toxic components of vine tea is exhibited in Table 1.

**TABLE 1:** Potentially toxic components of vine tea.

	Pubchem ID	Name	CAS ID	MeSH^®^ ID
1	161557	Dihydromyricetin	27200-12-0	C472036
2	5281672	Myricetin	529-44-2	C040015
3	439533	Taxifolin	480-18-2	C003377
4	5280863	Kaempferol	520-18-3	C006552
5	10639	Physcion	521-61-9	C008905
6	122850	Dihydrokaempferol	480-20-6	C080220
7	5280343	Quercetin	117-39-5	D011794
8	6072	Phloridzin	60-81-1	D010695
9	4788	Phloretin	60-82-2	D010693
10	72281	Hesperitin	520-33-2	C013015
11	72320	Nomilin	1063-77-0	C059405
12	5280450	Linoleic acid	2197-37-7	D019787
13	5281	Octadecanoic acid	57-11-4	C031183
14	985	Palmitic acid	57-10-3	D019308
15	5280934	Linolenic acid	463-40-1	D008042
16	6184	Hexanal	66-25-1	C010463
17	31289	Nonanal	124-19-6	C008664
18	5283321	(E, E)-2,4-heptadienal	4313-03-5	C502503
19	638014	Beta-ionone	14901-07-6	C008157
20	8063	Pentanal	110-62-3	C046012
21	454	Octanal	124-13-0	C031639
22	5283324	2-Octenal	2548-87-0	C057348
23	8175	Decanal	112-31-2	C021170
24	6549	Linalool	78-70-6	C018584
25	8186	Undecanal	112-44-7	C479548
26	61041	Safranal	116-26-7	C087963
27	17100	Alpha-terpineol	98-55-5	C016775
28	638011	Citral	5392-40-5	C007076
29	5283349	2,4-Decadienal	25152-84-5	C057349
30	4133	Methyl salicylate	119-36-8	C033069
31	8892	Hexanoic acid	142-62-1	C037652
32	8094	Heptanoic acid	111-14-8	C037652
33	379	Octanoic acid	124-07-2	C031492
34	8158	Nonanoic acid	112-05-0	C008776

### Potential Hepatotoxic Targets of the Toxic Components of Vine Tea

The 3D stereo structures of potential toxic components of vine tea were submitted to the PharmMapper server, and the system performed target prediction based on the principle of molecular reverse docking. The results returned 1,020 hit targets. After deleting nonhuman targets and duplicate values, a total of 551 potential targets were obtained, and annotating them as official names. A total of 699 targets related to hepatotoxicity, liver injury, and drug-induced liver injury were retrieved from the GeneCards database and CTD database. Fifty seven potential hepatotoxic targets of vine tea toxic components were obtained after intersecting with the action targets of vine tea toxic components.

### Protein–Protein Interaction Network

**FIGURE 1:**
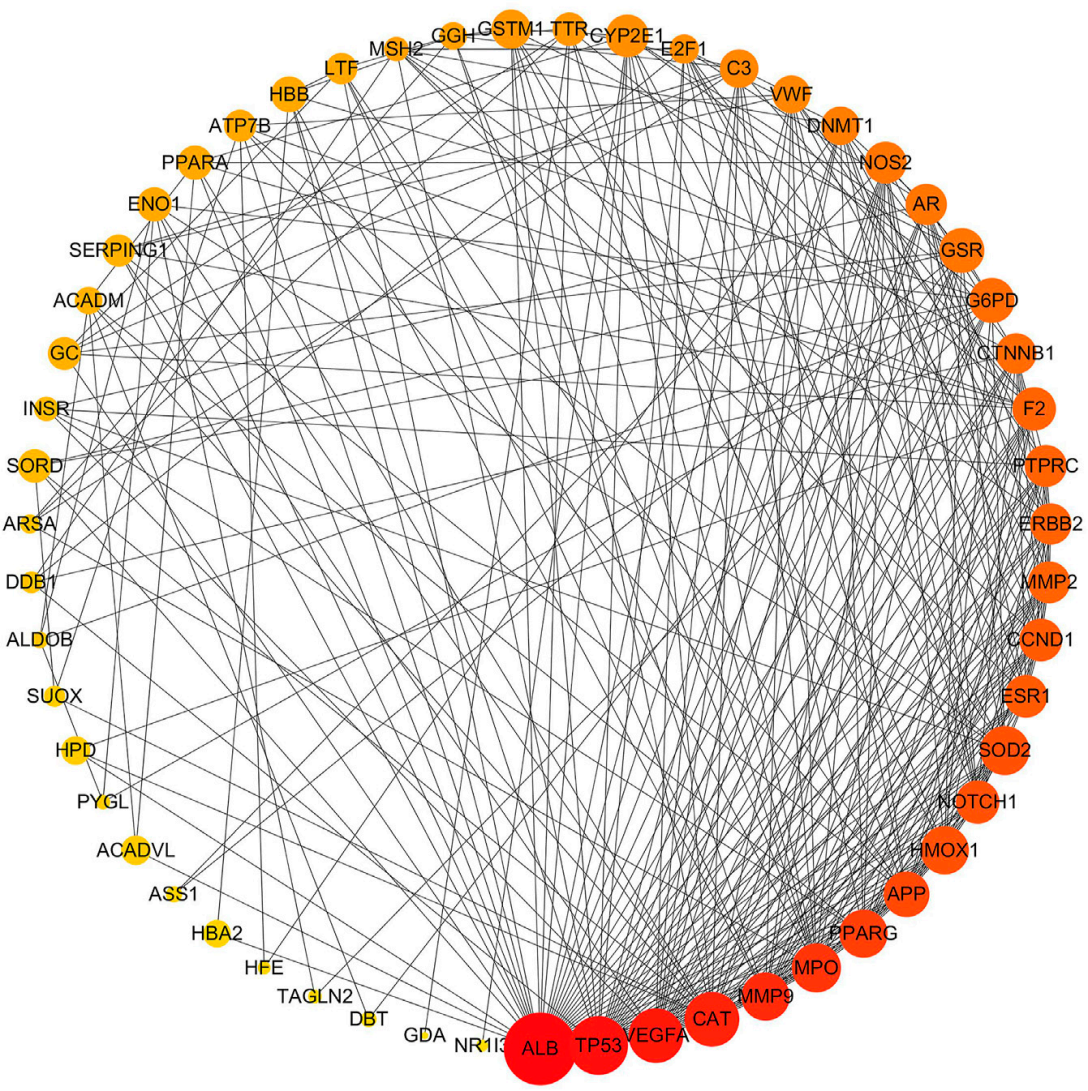
Hepatic toxicity targets protein–protein interaction network. Nodes are hepatotoxic targets; edges are target protein interactions.

Information on potential hepatotoxic targets of vine tea was submitted to the STRING database for analyzing protein–protein interactions (PPI). The topological properties of the PPI network allowed the analysis of the interaction mechanism of the potential hepatotoxic targets of vine tea. The results were imported into Cytoscape 3.6.1 software for visualization, as shown in Figure 1. The entire PPI network was constructed by 55 nodes and 308 edges. The node size was represented by the degree of closeness centrality, the node color was represented by degree, and the nodes of the entire network were ranked by the degree. The larger the node, the darker the color, the higher the ranking, the more important the node was in the network. The highest degree was ALB, which interacted with 38 targets (69%) in the network. Moreover there were also six interactive targets with more than 19 edges, namely TP53, VEGFA, CAT, MMP9, MPO, and PPARG. Those main functions are to regulate cell microenvironment, growth, proliferation, apoptosis, immunity, angiogenesis, and other physiological conditions, which are also important targets for the development of hepatotoxicity.

### Gene Ontology Enrichment Analysis and KEGG Pathway Enrichment Analysis

#### Gene Ontology Enrichment Analysis

**FIGURE 2:**
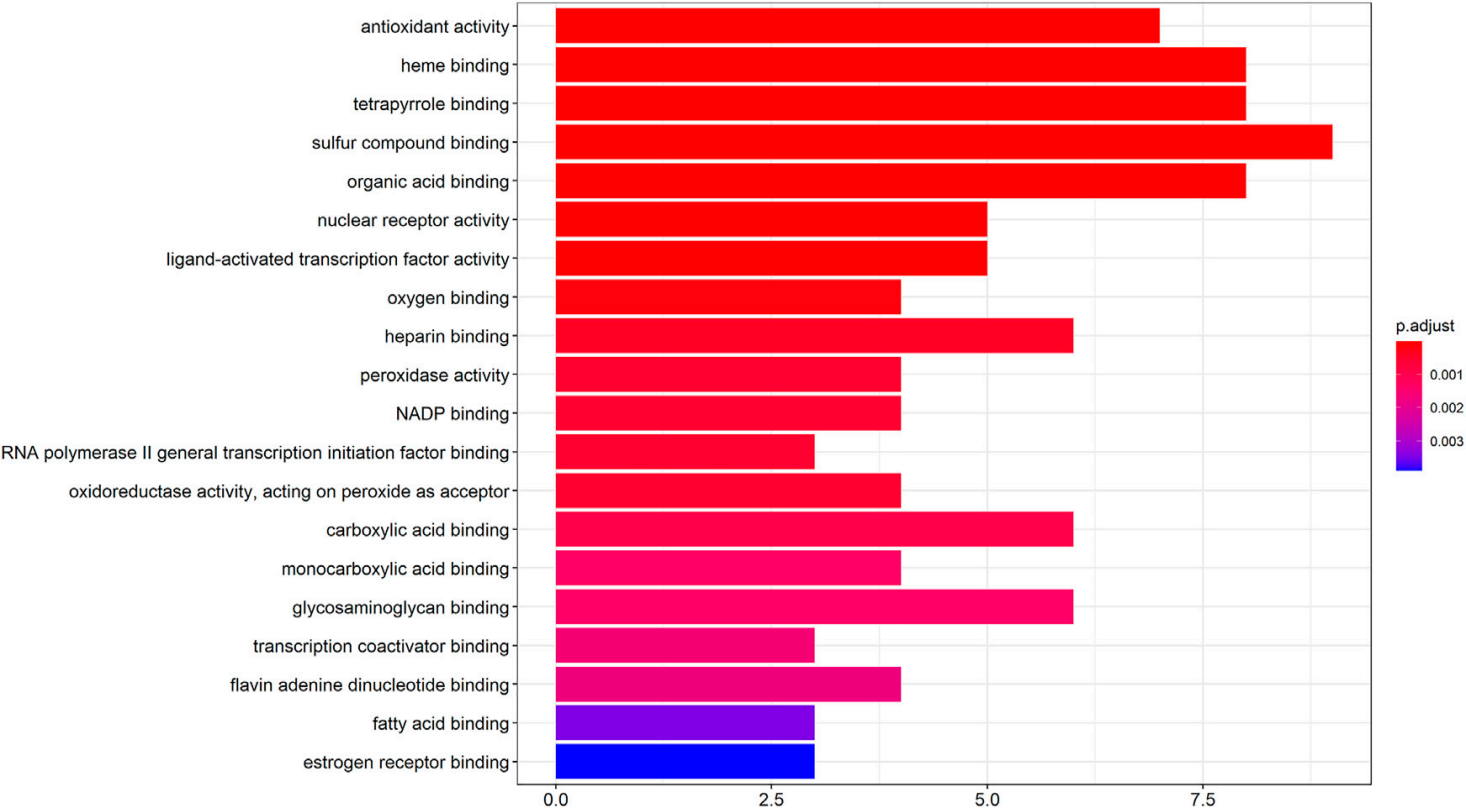
GO enrichment analysis of hepatotoxic target.

The GO molecular functions of the potential hepatotoxic targets of vine tea were enriched by the ClusterProfiler, setting the molecular function *p*-value cutoff to 0.05 and Q-value cutoff to 0.05. The top 20 GO molecular functions were plotted in a bar graph after enrichment. From Figure 2, it can be seen that there were 19 GO molecular functions with *p* < 0.001, and all of these molecular functions were related to drug-induced liver injury, among which there were nine molecular functions with ≥6 hits. The coenzyme binding with the highest number of targets (19%) may lead to drug-induced liver injury or cirrhosis by binding to pyridoxal phosphate, flavin adenine dinucleotide, and nicotinamide adenine dinucleotide. In addition, molecular functions, such as heme binding, organic acid binding, fatty acid binding, carboxylic acid binding, oxygen binding, and heparin binding, may result in toxicity and damage to the liver by affecting the metabolism of drugs or alcohol by the liver, inducing the incidence of negative blood-related effects (Dadarkar et al., 2011), lipid peroxidation (Das et al., 2010), and activating inflammation. Moreover, direct effects on antioxidant activity and nuclear receptor activity may cause an imbalance in hepatocyte microenvironmental homeostasis or damage by free radicals *in vivo* by decreasing the superoxide dismutase activity and mineralocorticoid receptor activity.

#### KEGG Pathway Enrichment Analysis

The Kyoto encyclopedia of genes and genomes (KEGG) pathway enrichment analysis was calculated by a Bioconductor package for potential hepatotoxic targets of vine tea with a *p*-value cutoff of 0.05 and Q-value cutoff of 0.05. The enrichment results are shown in Table 2. The enrichment results obtained 27 signaling pathways, including 13 cancer pathways, four cell proliferation or differentiation or apoptosis- related pathways, one oxidative stress pathway, one drug resistance pathway, four immunoloregulation related pathways, and three cell metabolism- related pathways. Among these signaling pathways, endocrine resistance, fluid shear stress and atherosclerosis, HIF-1 signaling pathway, longevity regulating pathway, thyroid hormone signaling pathway, hepatitis C, glutathione metabolism, carbon metabolism, and arginine biosynthesis were proved that these maybe the main pathways of hepatotoxicity in vine tea.

**TABLE 2:** KEGG enrichment pathway of hepatotoxic target genes.

Pathway ID	Pathway description	P.adjust	Count
hsa05219	Bladder cancer	8.7 × 10^−7^	7
hsa01522	Endocrine resistance	1.2 × 10^−5^	8
hsa05418	Fluid shear stress and atherosclerosis	9.5 × 10^−5^	8
hsa05215	Prostate cancer	9.5 × 10^−5^	7
hsa04066	HIF-1 signaling pathway	1.6 × 10^−4^	7
hsa05224	Breast cancer	9.4 × 10^−4^	7
hsa05205	Proteoglycans in cancer	9.4 × 10^−4^	8
hsa05216	Thyroid cancer	1.7 × 10^−3^	4
hsa01524	Platinum drug resistance	1.7 × 10^−3^	5
hsa05212	Pancreatic cancer	1.8 × 10^−3^	5
hsa05206	MicroRNAs in cancer	1.8 × 10^−3^	9
hsa04211	Longevity regulating pathway	3.3 × 10^−3^	5
hsa05213	Endometrial cancer	6.3 × 10^−3^	4
hsa05225	Hepatocellular carcinoma	7.7 × 10^−3^	6
hsa04919	Thyroid hormone signaling pathway	1.1 × 10^−2^	5
hsa05223	Non-small-cell lung cancer	1.1 × 10^−2^	4
hsa05167	Kaposi sarcoma-associated herpesvirus infection	1.3 × 10^−2^	6
hsa04610	Complement and coagulation cascades	1.8 × 10^−2^	4
hsa05210	Colorectal cancer	1.8 × 10^−2^	4
hsa05226	Gastric cancer	2.0 × 10^−2^	5
hsa05222	Small-cell lung cancer	2.1 × 10^−2^	4
hsa05160	Hepatitis C	2.3 × 10^−2^	5
hsa05165	Human papillomavirus infection	3.4 × 10^−2^	7
hsa00480	Glutathione metabolism	4.0 × 10^−2^	3
hsa01200	Carbon metabolism	4.4 × 10^−2^	4
hsa04213	Longevity-regulating pathway- multiple species	4.6 × 10^−2^	3

### Molecular Docking and Molecular Dynamics Simulation Analysis Results

**FIGURE 3:**
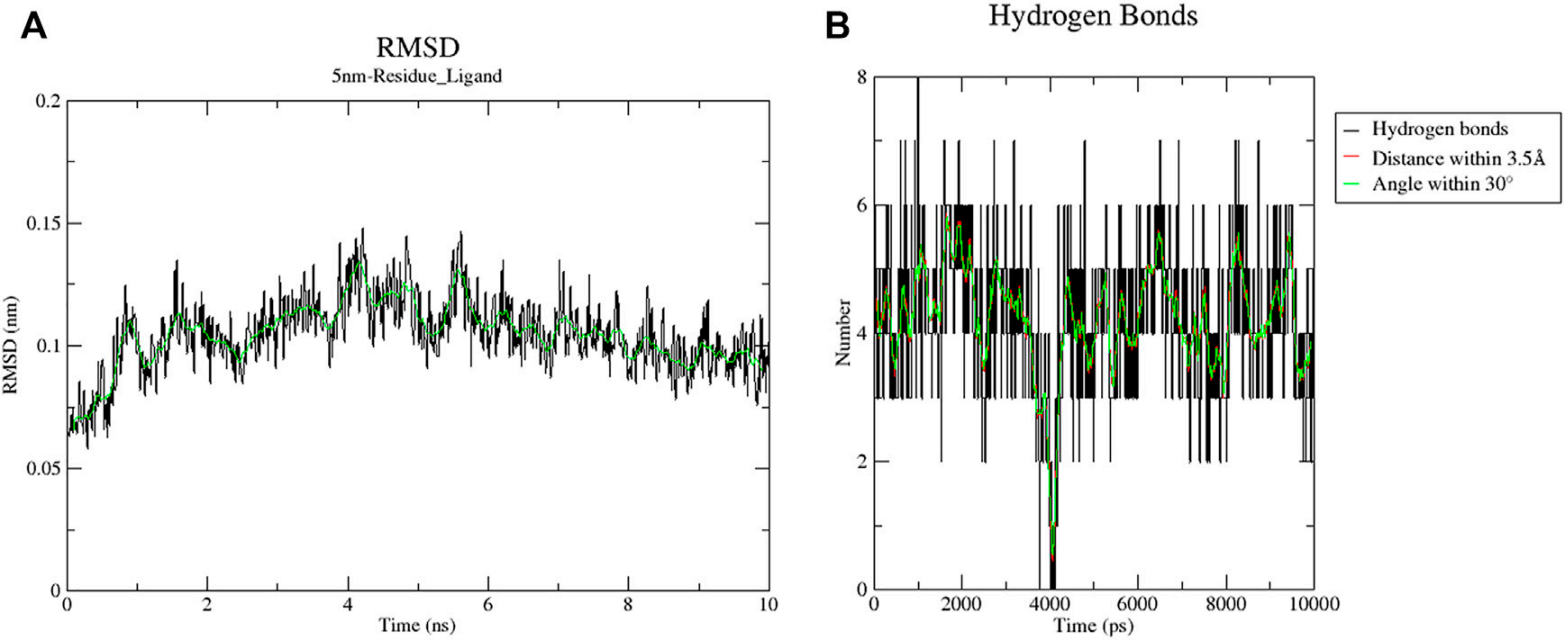
Results of 10 ns simulation of 1DGF and Phloridzin. Graph **(A)** indicates the RMSD variation of protein residue-ligand within 10 ns with the ligand as the center expanding 5 nm; graph **(B)** indicates the change in the value of hydrogen bonding generated by this ligand with protein residues within 10 ns.

Sixteen key target proteins of potential hepatotoxicity of vine tea were screened by the total conditions, and the corresponding protein crystal structures were obtained by the PDB database. Among them, nine protein crystal structures with natural ligands were extracted and pretreated for the docking task, and the specific docking parameter information is shown in Table 3. The RMSD of natural ligands and corresponding proteins after docking were all within 0.2 nm, indicating that the pretreatment and parameter settings before docking had a certain accuracy and could be used for the virtual docking task between the toxic components of vine tea and protein crystals. The 34 toxic components of vine tea and 16 protein crystals were docking calculated. The docking of 16 positive ligands with active effects to the corresponding proteins as a control, the docking results were represented by the scoring (kcal/mol), and the scoring equation included the sum of several effects such as electrostatic interaction, Van der Waals’ force, hydrogen bonding contributions, intermolecular conflicts, and intramolecular conflicts of the ligands. Generally speaking, a docking system score of ≥4.25 indicates that the ligand interacts with the protein, a score of ≥5.0 indicates that the ligand has some binding activity with the active region of the protein, and a score of ≥7.0 indicates that the ligand has strong binding activity with the active region of the protein (Hsin et al., 2013). The results in Table 4 lists the docking scores of key target proteins with positive ligands and vine tea toxic components (scored with an absolute value ≥6.0), while the docking scores not listed had 231 groups with an absolute value ≥4.25, 151 groups with an absolute value ≥5.0, and only eight groups with an absolute value ≥7.0. The entire docking task had 560 groups. Nearly half of the docking tasks indicated that the ligands interacted with the target proteins, mainly focusing on 15 vine tea toxicity components and 16 key target proteins, which further indicated that vine tea may manifest hepatotoxicity through multi-component-multi-target effects. Further analyzed in Table 4, among the vine tea toxic components whose absolute values of docking scores were greater than the positive ligands were mainly dihydromyricetin, hesperitin, kaempferol, myricetin, phloretin, phloridzin, quercetin, and taxifolin. Among them, the absolute values of the docking scores of phloridzin and 16 target proteins were all greater than 5.0, and they had strong binding effects with the active regions of seven target proteins. The energy minimum conformation of its docking with 1DGF was used as the input file of GROMACS software, and the results are shown in Figure 3 after molecular dynamics simulations of 10 ns. The RMSD variation of protein residues or protein residues-ligands within 10 ns of a 5 nm expansion centered on the ligand is shown in Figure 3A. It was obvious that the ligand was stable with the active binding region of the protein, its RMSD values were within 0.2 nm and were stable above or below 0.10 nm. Figure 3B shows the variation of the hydrogen bonding value between the ligand and the protein residue in 10 ns. Its average value was 4, the bond length was less than 0.35 nm, and the bond angle was in the range of 30°, indicating that the preprocessing and parameter setting of the simulation task was more reasonable. Moreover, it also showed that the results of the ligand–protein interaction and the docking task were in agreement within 10 ns, which verified the reliability of the LEDOCK program for this study.

**TABLE 3:** Molecular docking parameters.

Gene	Protein	PDB ID	Positive ligand Pubchem id	RMSD(Å)	Source ligand score (kcal/mol)
VEGFA	Vascular endothelial growth factor A	3BDY	56603655	—	—
CAT	Catalase	1DGF	15559239	0.7133	−9.45
MMP9	Matrix metalloproteinase-9	1GKC	54671203	0.7383	−6.69
PPARG	Peroxisome proliferator-activated receptor	3NOA	3651377	0.3112	−10.02
HMOX1	Heme oxygenase 1	6EHA	4971	0.3605	−5.94
NOTCH1	Neurogenic locus notch homolog protein 1	2F8X	43815	—	—
SOD2	Superoxide dismutase [Mn], mitochondrial	1ZTE	29327	—	—
ESR1	Estrogen receptor	5ACC	104741	0.2131	−7.25
CCND1	G1/S-specific cyclin-D1	2W96	119307	—	—
MMP2	72 kDa type IV collagenase	1RTG	23310222	—	—
NOS2	Nitric oxide synthase, inducible	4N0S	2146	0.4876	−3.41
CTNNB1	Catenin beta-1	1P22	40486	-	-
AR	Androgen receptor	3RLJ	67171867	0.3687	−7.87
DNMT1	DNA (cytosine-5)-methyltransferase 1	3SWR	702558	0.431	−7.98
ERBB2	Receptor tyrosine-protein kinase erbB-2	3RCD	5280343	0.3687	−8.97
TP53	Cellular tumor antigen p53	5BUA	6450226	—	—

**TABLE 4:** Molecular docking of potentially toxic components of vine tea.

PDB ID	Pocket size (Xmin X_max_, Y_min_ Y_max_, Z_min_ Z_max_)	Pubchem id	Score (kcal/mol)
3BDY	-55.137 -43.843,-63.747 -51.128,-6.532 6.32	56603655	−6.12
1DGF	17.059 37.69,65.515 85.329,60.61 78.625	15559239	−5.71
		72281	−6.21
		5281672	−6.22
		6072	−8.42
1GKC	57.951 73.263,23.793 38.373,108.689 126.996	54671203	−5.88
		161557	−6.09
		5281672	−6.43
		6072	−7.22
		5280343	−6.14
3NOA	-17.606 -1.512,-1.912 13.828,27.491 52.487	3651377	−5.17
		161557	−6.26
		5280863	−6.07
		5281672	−6.31
		6072	−7.74
		5280343	−6.35
		439533	−6.29
6EHA	8.197 22.295,-8.896 6.105,25.248 42.982	4971	−4.75
		4788	−6.35
		5280343	−6.12
2F8X	79 103,51 85,-22 14	43815	−6.55
		122850	−6.06
		161557	−6.55
		5280863	−6.12
		5281672	−6.57
		4788	−6.45
		6072	−8.63
		5280343	−6.22
		439533	−6.21
1ZTE	26 46,-1.1 16.9,51 67	29327	−5.49
		6072	−7.04
5ACC	5.996 22.284,13.209 31.381,57.414 75.909	104741	−6.36
		6072	−6.66
2W96	-1 25,-10 16,19 45	119307	−4.56
1RTG	10 30,20 36,7.5 21.5	23310222	−5.2
		6072	−6.4
4N0S	-22.432 -7.127,5.408 20.213,34.982 46.184	2146	−3.15
		6072	−6.8
1P22	-15 5,18 38,-23 -3	40486	−6.79
		72320	−6.13
		6072	−6.97
3RLJ	19.021 34.81,-4.706 11.518,-0.91 16.722	67171867	−8.34
		161557	−6.7
		72281	−6.03
		5280863	−6.03
		5281672	-6.14
		4788	-6.6
		6072	−6.16
		5280343	−6.5
		439533	−6.32
3SWR	-12.867 2.786,-10.553 8.655,23.049 40.581	702558	−6.64
		161557	−6.78
		5281672	−6.65
		4788	−6.38
		6072	−7.95
		5280343	−6.62
3RCD	2.899 22.061,-7.118 13.045,19.662 36.368	5280343	−5.99
		161557	−6.06
		5281672	−6.45
		6072	−7.06
5BUA	91.7 111.7,69.5 89.5,-24.9 -4.9	6450226	−3.89
		6072	−6.37

### Transcriptome Analysis Results

**FIGURE 4:**
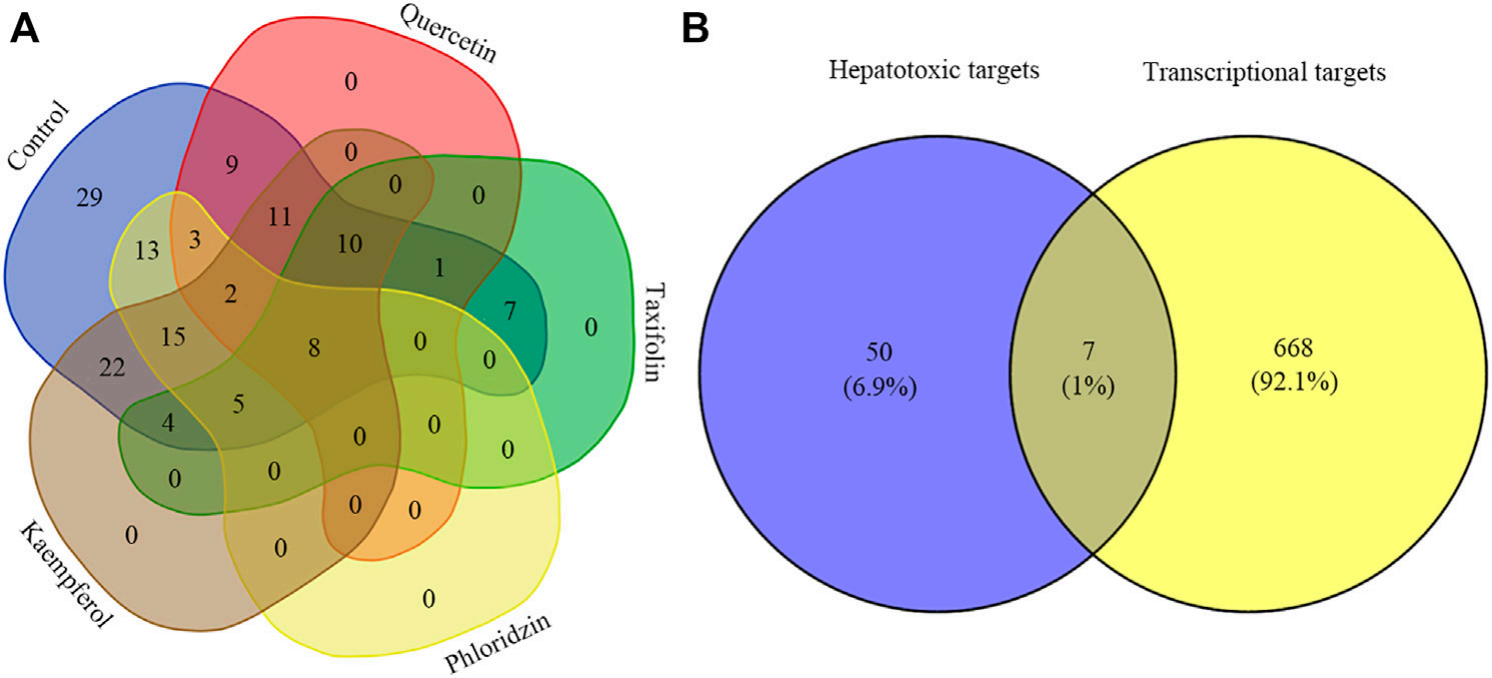
Venn diagram of transcriptome analysis. Graph **(A)** indicates the distribution of GO functions enriched by vine tea components as perturbation factors similar to the GO functions enriched by the reference expression profile; graph **(B)** indicates the intersection of transcriptional targets hit by hepatotoxic targets and liver injury-related GO functions.

The results of the aforementioned analysis obtained eight major toxic components of vine tea. When these components were used as perturbation factors to search the GEO database for relevant expression profiles, only a few components’ expression profiles such as quercetin, taxifolin, phloridzin, and kaempferol were found, whose database platform numbers were GSE62805, GSE59704, GSE38138, and GSE145665, respectively. At the same time, referring to the expression profile no. GSE5789, the experimental protocol for this data was obtained with the transcriptional data using toxic drugs (2,3,7,8-tetrachlorodibenzo-p-dioxin, 3,3′,4,4′,5-pentachlorobiphenyl, 2,2′,4,4′,5,5′-hexachlorobiphenyl, and 2,3,4,7,8-pentachlorodibenzofuran) as perturbers that caused liver damage. After the expression profiles were enriched for the GO functional biological process (BP) modules separately, the BP modules corresponding to the enrichment of vine tea components were compared with the BP modules enriched for the reference expression profiles for similarity. Eight similar common BP modules were found, namely gland growth, angiogenesis, morphogenesis of blood vessels, epithelial cell differentiation, development of circulatory system, sprouting angingenesis, and muscle organ growth, which were more consistent with the gene functions of the important targets analyzed previously, and the results are shown in Figure 4A. The BP modules associated with liver injury were further analyzed, and the gene sets hit by the BP modules of expression profiles and the corresponding similar BP modules of various components were compared for similarity. The gene sets of GO functional similar modules with correlation coefficients greater than 0.6 were found to have a functional similarity in the range of 0.6–0.9, indicating high similarity between the BP modules enriched by the transcriptional expression of vine tea components for the treatment of perturbation factors and the characteristics of transcriptional expression of liver injury, and the results are represented in Table 5. This revealed that these four vine tea components may promote biological processes at the cellular level such as the response to reactive oxygen species, response to toxic components, response to oxidative stress, lipid metabolism, and apoptosis. Subsequently, the genes hit by these highly similar modules were intersected with the hepatotoxic targets obtained previously, which obtained seven common target genes, such as HMOX1, PPARG, PTPRC, G6PD MMP9, MMP2, and GSTM1, as shown in Figure 4B. Further validating the reliability of the screening of the aforementioned key targets, and suggesting that vine tea components played an important role in angiogenesis, cell proliferation, and cell differentiation.

**TABLE 5:** Gene set correlation and hit count of GO similar functional modules.

Compound	GO ID	Description	Relativity	Counts
Quercetin	GO:0000302	Response to reactive oxygen species	0.745	33
	GO:0034614	Cellular response to reactive oxygen species	0.721	21
	GO:0010035	Response to inorganic substance	0.793	50
	GO:0034097	Response to cytokine	0.78	50
	GO:0071407	Cellular response to organic cyclic compound	0.727	26
	GO:0009636	Response to toxic substance	0.746	50
	GO:0071363	Cellular response to growth factor stimulus	0.741	26
	GO:0070848	Response to growth factor	0.783	50
	GO:0098754	Detoxification	0.667	50
	GO:0044344	Cellular response to fibroblast growth factor stimulus	0.702	26
Taxifolin	GO:0009611	Response to wounding	0.747	60
	GO:0006979	Response to oxidative stress	0.77	64
	GO:0071900	Regulation of protein serine/threonine kinase activity	0.619	5
	GO:0043069	Negative regulation of programmed cell death	0.608	8
	GO:0060548	Negative regulation of cell death	0.636	11
	GO:0070997	Neuron death	0.675	12
	GO:1901215	Negative regulation of neuron death	0.669	11
	GO:0043524	Negative regulation of the neuron apoptotic process	0.653	10
	GO:0043523	Regulation of the neuron apoptotic process	0.668	12
Phloridzin	GO:0010035	Response to inorganic substance	0.656	10
	GO:0071407	Cellular response to organic cyclic compound	0.787	41
	GO:0009611	Response to wounding	0.665	17
	GO:0033993	Response to lipid	0.768	24
	GO:0071902	Positive regulation of protein serine/threonine kinase activity	0.718	11
	GO:0071900	Regulation of protein serine/threonine kinase activity	0.71	11
	GO:0043066	Negative regulation of the apoptotic process	0.804	34
	GO:1901568	Fatty acid derivative metabolic process	0.709	72
	GO:0006690	Icosanoid metabolic process	0.638	6
	GO:0043065	Positive regulation of the apoptotic process	0.758	23
	GO:0043523	Regulation of the neuron apoptotic process	0.745	23
Kaempferol	GO:0000302	Response to reactive oxygen species	0.85	62
	GO:0071407	Cellular response to the organic cyclic compound	0.856	97
	GO:0009611	Response to wounding	0.848	75
	GO:0006631	Fatty acid metabolic process	0.676	31
	GO:0006979	Response to oxidative stress	0.864	75
	GO:0071345	Cellular response to cytokine stimulus	0.908	144
	GO:0071900	Regulation of protein serine/threonine kinase activity	0.712	13
	GO:0043066	Negative regulation of the apoptotic process	0.678	6
	GO:0051402	Neuron apoptotic process	0.696	7
	GO:0071902	Positive regulation of protein serine/threonine kinase activity	0.729	13
	GO:0044255	Cellular lipid metabolic process	0.708	38
	GO:0071363	Cellular response to growth factor stimulus	0.911	128
	GO:0070848	Response to growth factor	0.91	128
	GO:0080135	Regulation of cellular response to stress	0.84	34
	GO:0043065	Positive regulation of the apoptotic process	0.686	6
	GO:0002237	Response to the molecule of bacterial origin	0.883	85
	GO:0043523	Regulation of the neuron apoptotic process	0.689	6

## Discussion

In this study, we analyzed the GO molecular functions and KEGG pathway of the same targets by integrating the action targets of the toxic components of vine tea and the hepatotoxicity-related targets and validated them by molecular docking techniques. By mining the expression profile of GEO, we analyzed the toxicological effects of the GO functional modules enriched by the transcriptional expression of vine tea components as perturbation factors at the cellular level, to predict the potential hepatotoxic action mechanism of vine tea. The liver is involved in the physiological processes of body metabolism, hormone balance, and toxin removal, and is an essential organ of the human body. Most drugs are absorbed orally through the gastrointestinal tract and then metabolized by the liver and excreted from the kidneys; if a few drugs are metabolized by the liver to form end-toxicants or transient active substances, then the drugs are exposed to the liver will lead to pharmacologic liver damage. Therefore, the active components of drugs should be actively concerned whether they can cause hepatotoxicity while exerting their medicinal effects, and some active components may cause hepatotoxicity through direct cellular toxicity, oxidative stress, immune response, and cellular metabolism (Bleibel et al., 2007). Although some drugs are relatively safe, they may also cause liver injury (Galan et al., 2005). There are reports in the literature that Chinese herb medicines can cause liver injury (S et al., 2014). Earlier literature (Yang et al., 2006) demonstrated that the consumption of biological doses of vine tea was safe through safety toxicology experiments in rats, however, the available literature (Sahu and Gray, 1996; Dzoyem et al., 2013; Skibola and Smith, 2000; Galati and O'Brien, 2004) showed that at certain doses, durations, and individual differences in the effects of vine tea, kaempferol, quercetin, myricetin, taxifolin, dihydromyricetin, physcion, dihydrokaempferol, phloretin, and other components can induce mutagenic cytotoxicity.

The role of chemical components such as flavonoid substances and polyphenols in vine tea *in vivo* is complex. Although there is a large body of literature supporting the antioxidant function of dietary polyphenol or flavonoid components *in vivo*, the antioxidant activity of these substances *in vivo* can be expressed by the substances themselves either directly as reactive oxygen radical scavengers or indirectly as regulators of intracellular antioxidant enzymes and pro-oxidant enzymes, when involved in peroxidases containing systems with redox metals, these substances have been shown to act as pro-oxidants, directing the formation of reactive oxygen radicals (ROS) and phenoxyl radicals, further promoting the destruction of lipids, DNA, or their biomolecules (Decker, 1997), and increasing the concentrations of substances, which can mediate the breakage of cellular DNA and activate the cellular caspase apoptotic pathway (Hadi et al., 2000), leading to non-specific necrosis of cells and cause cytotoxicity. For example, quercetin, a toxic component of vine tea with a catechol B ring, is absorbed orally by the body and can be distributed to liver organs (de Boer et al., 2005), where it is oxidized and metabolized to o-quinone by tyrosine oxidase and catalase in hepatocytes (Oyama et al., 1999) and bound to macromolecules as an electrophilic body, and may also react with O_2_ redox to generate O_2_—or OH radicals, and these oxidized quinones or quinone methyl intermediates may also react with glutathione to form adducts (Galati et al., 2001) when the resulting cytotoxicity is related to the quercetin-dependent concentration. It has also been shown that the opposite occurs, as ROS in hepatocytes metabolizes flavonoids such as quercetin into products that covalently bind to cellular DNA and proteins (Walle et al., 2003), and similarly, kaempferol and myricetin has been shown to cause ROS formation in systems containing peroxidases involved in the presence of redox metals (Galati and O'Brien, 2004). In contrast, the transcriptional analysis indicated that four components of vine tea characterize the cellular response to reactive oxygen species, response to oxidative stress, response to toxic substances, and BP function in response to injury at the cellular level, which is consistent with the aforementioned analysis.

Another mechanism of cytotoxicity that causes apoptosis is that quercetin and myricetin directly affect the mitochondrial membrane potential in a pro-oxidant effect with the involvement of transition metals (Said Ahmad et al., 1992), and this effect leads to the rupture of the mitochondrial membrane and the release of cytochrome C and triggers the release of mitochondrial toxicity, which is an irreversible marker of cellular mortality, and that the toxic components of vine tea, represented by palmitic acid, represented by saturated fatty acids, induces lipid apoptosis (Jinju et al., 2016). The present work demonstrated that the interaction of vine tea toxic components with superoxide dismutase (SOD2) and catalase (CAT) may affect the antioxidant activity of hepatocytes and disrupt the intracellular balance of scavenging toxic substances such as superoxide anion radicals, indirectly leading to the accumulation of toxins and attacking intracellular biomolecules and inducing apoptosis pathway. In contrast, 11 (32.35%) of the vine tea toxic components act on superoxide dismutase and 15 (44.12%) on catalase, so vine tea may affect the activity of superoxide dismutase and catalase downstream of the longevity regulating pathway, reducing the detoxification capacity of hepatocytes and increasing the potential of toxins to damage cells. The transcriptional analysis revealed that the enriched BP module was highly similar in the regulation of the apoptotic process, cytokine stimulation response, and regulation of protein serine/threonine kinase activity, which verified that the characteristics of this component as a perturber- induced cell expression were consistent with the behavior of the apoptotic mechanism.

In addition, this study demonstrated that vine tea toxic components can act on heme oxygenase 1 (HMOX1), glutathione S-transferase Mu 1 (GSTM1), whose involvement in phase II metabolism is considered as a pathway for cellular clearance of exogenous toxic substances, and it is important to note that inhibition of these enzymes may lead to accumulation of exogenous toxic substances. Among them affecting the glutathione metabolic pathway can lead to depletion of glutathione, thus affecting the content of components such as bilirubin and glutathione, which is consistent with the literature reporting that flavonoids cause glutathione oxidation in rat hepatocytes (Galati et al., 2002). On the other hand, glutathione S-transferase P is closely linked to CYP450 in the signaling pathway for the metabolic action of exogenous compounds, and the CYP enzyme (CYP2E1) acting on toxic components of vine tea is involved in molecular functions such as heme binding, tetrapyrrole binding, sulfide binding, oxygen binding, NADP binding, and oxidoreductase activity, and the functions of the transcriptome- enriched BP module in cellular detoxification, reaction to inorganic and organic substances, fatty acid and lipid metabolism indicate that the toxic components of vine tea can perturb cellular bio metabolism and may affect the metabolism of exogenous compounds in hepatocytes and prolong the duration of pharmacological action of toxic components and bioactive substances of vine tea, increasing the chance of Drug-drug interaction (DDI), which in turn causes some toxic side effects in the liver (Jana and Paliwal, 2007). Here, it should be noted that the physical differences of different people make the specific reactions triggered by the differences in the expression and catalytic activity of metabolic enzymes such as CYP enzymes in sensitive individuals, and most of them can tolerate the dose and duration of drugs well, only for a very small number of physically sensitive healthy individuals hide dangerous or even fatal signals. Both network toxicology and transcriptomic analyses support the significant relevance of vine tea components in angiogenesis and cell differentiation through the interactions of vascular endothelial growth factor A (VEGFA), neurogenic locus notch homolog protein 1 (NOTCH1), 72 kDa type IV collagenase (MMP2), catenin beta-1 (CTNNB1), receptor tyrosine-protein kinase erbB-2 (ERBB2), and other proteins interact with each other, whether this effect predicts that the toxic components of vine tea can block bile entry into the bile ducts and cause bile stasis by affecting vascular and peripheral capillary vessels is to be verified by further experiments. In conclusion, the types of liver damage induced by drugs vary, depending on the nature, dosage, and duration of the drug itself, and the toxic components of vine tea have both significant pharmacological activity and potential for cytotoxicity, so it is of some significance to explore the mechanism of hepatotoxic effects of vine tea in depth.

 In summary, this work demonstrates that vine tea has a multi-component, multi-target, and multi-pathway interrelationships and that the flavonoid-based toxic components of vine tea may promote oxidative stress and pro-oxidative generation of free radicals and attack the mitochondria at the cellular level, causing cytotoxicity, triggering apoptotic mechanisms, or affecting the metabolic activities of cells. These adverse effects are dose-dependent toxic manifestations, and although the general population has a good degree of tolerance at certain doses and durations, the potential pitfalls hidden in a few healthy individuals with sensitive constitutions should not be overlooked. Such herb tea beverages do not require SFDA approval for market circulation, and their therapeutic functions and taste are increasingly favored by consumers worldwide, and the assessment of potential toxicity and drug interactions has not been definitional, so mixing and abuse with other herbs should be avoided, and it is recommended that a safe dosage standard for consumption of herbal-based tea beverages be established. This work is based on database mining and analysis to investigate whether vine tea is potentially hepatotoxic using a network toxicology and molecular dynamics approach. Although the method is becoming increasingly popular, its accuracy and reliability are related to the subjective choice of the user, together with drawbacks such as the limitations of the database, which require the user to have a deep basic knowledge of network toxicology and molecular dynamics theoretical approach. However, it also illustrates the important contribution of the method to the development of biology, which will become a core research technique in modern biology and further generate new interdisciplinary disciplines. Finally, the metabolic indexes and action targets predicted in this paper are informative but do not fully reflect the overall profile of vine tea. Because of the potential applications in material and pharmaceutical sciences for constituents containing therapeutic functions such as dihydromyricetin, vine tea is of increasing interest to researchers. Further follow-up studies and validation of the toxicological effects, serum pharmacology, and serum medicinal chemistry of various constituents of vine tea are essential to guide future product development and the possibility of reducing the cost of new formulations.

## Data Availability

The datasets supporting the conclusions of this article are available in a public database from PubChem, SMILSS, CTD, PharmMapper, UniProt, GeneCards, STRING, GEO. The accession numbers of gene chip are GSE62805, GSE59704, GSE38138, GSE145665, GSE5789.
